# Sodium Fluorescein-Guided Resection under the YELLOW 560 nm Surgical Microscope Filter in Malignant Gliomas: Our First 38 Cases Experience

**DOI:** 10.1155/2017/7865747

**Published:** 2017-10-16

**Authors:** Ningning Zhang, Hailong Tian, Dezhang Huang, Xianbing Meng, Wenqiang Guo, Chaochao Wang, Xin Yin, Hongying Zhang, Bin Jiang, Zheng He, Zhigang Wang

**Affiliations:** Department of Neurosurgery, Qilu Hospital, Qingdao, China

## Abstract

**Objective:**

Sodium fluorescein (FL) had been safely used in fluorescence-guided microsurgery for imaging various brain tumors. Under the YELLOW 560 nm surgical microscope filter, low-dose FL as a fluorescent dye helps in visualization. Our study investigated the safety and efficacy of this innovative technique in malignant glioma (MG) patients.

**Patients and Method:**

38 patients suffering from MGs confirmed by pathology underwent FL-guided resection under YELLOW 560 nm surgical microscope filter. We retrospectively analyzed the clinical characters, microsurgery procedure, extent of resection, pathology of MGs, progression-free survival (PFS), and overall survival (OS).

**Results:**

Thirty-eight patients had MGs (10 WHO grade III, 28 WHO grade IV). With YELLOW 560 nm surgical microscope filter combined with neuronavigation, sodium fluorescein-guided gross total resection (GTR) was achieved in 35 (92.1%) patients and subtotal resection in 3 (7.69%). The sensitivity and specificity of FL were 94.4% and 88.6% regardless of radiographic localization. Intraoperatively, 10 biopsies (10/28 FL[+]) showed “low” or “high” fluorescence in non-contrast-enhancement region and are also confirmed by pathology. Our data showed 6-month PFS of 92.3% and median survival of 11 months.

**Conclusion:**

FL-guided resection of MGs under the YELLOW 560 nm surgical microscope filter combined with neuronavigation was safe and effective, especially in non-contrast-MRI regions. It is feasible for improving the extent of resection in MGs especially during emergency cases.

## 1. Introduction

According to World Health Organization (WHO), malignant glioma (MG) is defined as grade III or IV glioma. It is a highly invasive tumor type and has demonstrated a poor prognosis rate even with the use of current treatment modalities such as microneurosurgery, chemotherapy, and radiotherapy. Nevertheless, maximal extent of resection improved the progression-free survival (PFS) and overall survival (OS) of patients who were suffering from MGs [1, 2]. The main purpose to treat the patients includes safe and maximal resection of MG, while ensuring neurological integrity. Several innovative techniques of neurosurgery have been attempted to improve the extent of resection, such as neuronavigation [3], intraoperative ultrasound [4], and intraoperative MRI. But none of these treatment modalities resolved the perfect resection of glioma cells of MGs exactly from normal brain tissue in the ideal conditions.

In pursuit of real-time intraoperative guidance method for the safe and maximal resection of tumors, fluorescence-guided surgery has emerged as an advanced adjunctive technique, which had documented increase in the extent of tumor resection. 5-Aminolevulinic Acid (5-ALA) is an intermediate metabolite of heme biosynthesis, which is transformed to protoporphyrin IX (PpIX) and is accumulated in MG cells and can be visualized under special filter. Although accumulated PpIX in glioma cells theoretically could discriminate malignant glioma tissues from normal brain tissues, few other studies have described that “bleaching” of 5-ALA-fluorescent dye obscured the tumor boundary [5]. PFS was greater than 6 months among patients who underwent 5-ALA-guidance than white light [6]. Furthermore, 5-ALA fluorescence-guided surgery has other shortcomings such as drug's phototoxicity, extortionate price, and not being approved by Food and Drug Administration, which limited its widespread application.

Due to the above limitations, sodium fluorescein- (FL-) guided surgery recently had been paid more attention by neurosurgeons than 5-ALA. FL is an easily available and biosafe fluorescein dye with a peak excitation at 465 to 490 nm and emission between 500 and 550 nm and has been used extensively and safely for many years especially in ophthalmology [7, 8]. Moore et al. attempted to obtain the value of diagnosis of FL in intracranial tumor and described the use of FL for the first time in 1947 [9, 10]. FL disseminates through disrupting the blood-brain barrier (BBB) and accumulates in the extracellular space of brain tumors; otherwise, FL leaks into the normal brain tissues minimally keeping the BBB intact and is readily cleared. Thus, FL-guided surgery has been discussed by many studies to improve the extent of resection, which in turn look forward to improve the prognosis of MG. Gross tumor resection was significantly increased with FL-guided surgery, even though high doses (>20 mg/kg) of FL were employed in the resection of glioblastomas through traditional microscope [5, 11]. Low doses (5–10 mg/kg) of FL had also been documented to be effective and safe adjunctive technique for high volume of mean tumor resection (90.5%) [12]. A newly advanced microscope, PENTERO 900 with a special filter (YELLOW 560 nm), was used at very low doses (3-4 mg/kg) of FL [13]. This may in turn prevent potential risk of dose-dependent side effects, in spite of the fact that it had been covered by very few literature studies with high-dosage use [14, 15]. We exhibited our primary experience and results of FL-guided resection of MGs from January 2014 to December 2016 in neurosurgery department of Qilu Hospital (Qingdao) of Shandong University. With the usage of YELLOW 560 nm surgical microscope filter, the study aimed to provide more evidence to the effectiveness and usefulness of FL-guided surgery.

## 2. Patients and Methods

Our retrospective study inclusion criteria were similar to the previous study criteria of FLUOGLIO trial by Acerbi et al. [16], which were as follows: (1) age 18 to 75 years; (2) newly diagnosed, untreated, or recurrent MGs (based on the preoperative brain MRI with and without contrast as reported by Acerbi and confirmed by pathological reports postoperative); (3) tumor location allowing for gross resection of contrast-enhancing area as determined by the surgeon and neuroradiologist. The exclusion criteria were as follows: (1) age < 18 years or >75 years; (2) a tumor that is originated in the brainstem; (3) the presence of a non-contrast-enhancing area, suggesting a low-grade glioma with malignant transformation; (4) medical reasons precluding MRI with contrast-enhancement (CE); (5) renal insufficiency; (6) hepatic insufficiency; and (7) active malignant tumors at any other part of the body. This study included only patients with WHO grade III and glioblastoma multiforme (GBM). Informed consent was obtained before operation from all the patients who underwent FL-guided surgery. This study was approved by the ethic committee of Qilu Hospital.

Clinical assessment included a preoperative estimate within 7 to 10 days of surgery and a postoperative appraisal within the first 24 to 72 hours after surgery. MRI studies with 3.0-T Philips Ingenia scanner with and without gadolinium-enhancement were obtained from all patients. Preoperative MRI studies were performed within 7 days of surgery and postoperative studies within the first 24 to 72 hours after surgery in all cases. The tumor and residual volume were calculated preoperatively and postoperatively using open-source software (Osirix for Macintosh, http://www.osirix-viewer.com/). The area of gadolinium-enhancement greater than 0.175 cm^3^ by a calculated volume was considered to be residual tumor [6]. Postoperatively, the patients were disposed according to the Stupp protocol (i.e., postoperative radiosurgery and chemotherapy) with radiotherapy (25 cases) and concomitant temozolomide (23 cases) therapy [17].

After the induction of anesthesia and before skin incision, 2-3 mg/kg bodyweight (3 mg/kg in first 28 cases and 2 mg/kg in late 10 cases) of 20% solution of sodium fluorescein (Guangzhou Baiyun Shan Ming Xing Pharmaceutical Company, China, National Drug number: H44023400) diluted to 1% was administered by bolus intravenous injection. Pentero 900 microscope (with fluorescence kit, YELLOW 560, Carl Zeiss Meditec, Oberkochen, Germany) was employed during the neurosurgery. YELLOW 560 is a new fluorescence kit, described in previous studies [13, 16], designed at an excitation wavelength of 460 to 500 nm and for observation at 540 to 690 nm, respectively. This in turn reduces the fluorescein dosage to 3 mg/kg according to the research performed in current studies [13, 18, 19]. Hence, FL-guidance at a dose of 3 mg/kg was employed in the first 22 cases, and in addition, we reduced the dosage to 2 mg/kg from February 2016 and obtained the same effective imaging results (late 10 cases). Neuronavigation (BrainLab) based on T1-weighted gadolinium-enhancement MRI was used for designing the surgical plan and ascertained the biopsies that demonstrated margin localization between the tumor and normal tissues but not judged for extent of resection. Intraoperative gross total resection (GTR, may be considered as aggressive or super-total resection according to postoperative T1-weighted CE MRI) was judged only by neurosurgeon when all the fluorescein dyed tissues were removed, except for one case which involved the ganglia region and in turn depended on the intraoperative neuronavigation. Two cases encompassed middle cerebral artery (MCA) by tumor dependent on neurosurgeon's judgement. Neurophysiological monitoring was employed when tumors were located in or near to the eloquent areas based on the details described by Cordella study [20]. With the YELLOW 560 filter, fluorescent signals indicated tumor tissues and the nonfluorescent tissues indicated normal tissues and could be visualized distinctively in real-time. Most of the surgical time, the YELLOW 560 filter was used by the neurosurgeon; only when it was necessary to coagulate hemostasis using bipolar or get the biopsy specimens, the microscope could be switched to white light illumination easily by pressing handgrip switch button. To avoid the blurring visualization, neurosurgeons tried their best to suck the operative field bleeding by an aspirator. Sometimes, tumor was removed in an inside-out fashion by ultrasonic aspiration until FL-guided tissue was removed completely.

Follow-up examinations on clinical assessment patterns covered most of the outpatient clinical follow-up, intermittent inpatient clinical follow-up, telephone follow-up, cell phone message follow-up, interview follow-up, WeChat or Tencent QQ follow-up, and mailbox follow-up. Radiological assessment was obtained within 1 month after the end of neurosurgery and then 3 months and then every 6 months based on outpatient clinical follow-up and intermittent inpatient clinical follow-up only. The estimated clinical data incorporated the same evaluations and were performed at the preoperative and postoperative appraisement duration of hospitalization. Imaging follow-up was performed by incorporating with the MRI with and without gadolinium-enhancement (Philips Ingenia 3.0 T). The occurrence of new gadolinium-enhancing area of MRI greater than 0.175 cm^3^ was defined as progression based on FLUOGLIO [16] and RANO criteria [21]. The side effects involved during the administration of fluorescein after general anesthesia such as hypotension, seizures, bronchospasm, or anaphylaxis could be estimated for objective findings.

Biopsy specimens were obtained and then labeled as “none,” “low,” and “high” density based on the surgeon's visualization from real-time intraoperative microscope. “Positive” FL (+) was defined as “low” and “high” density tissues, while “negative” FL (−) was defined as “none” density tissues. Biopsy tissues were fixed in 10% formalin and embedded in paraffin for standard pathological analysis. One-8 random biopsy specimens from each patient were obtained at the tumor margin areas, which was defined by FL and T1-weighted CE neuronavigation guidance. Consequently 89 biopsy specimens were obtained to calculate the sensitivity and specificity, and we also documented the correlation between FL-guided tumor margin and T1-weighted CE MRI margin. Tumor core biopsies were not included for our purpose of study. In these specimens, histological analysis was performed to discriminate between tumor tissue (including blank tumor and peritumoral area [gliosis or tumor cell infiltration]) and nontumor tissue; GFAP (glial fibrillary acidic protein) and Ki 67 (MIB-1, for the proliferation index) were also performed by immunohistochemistry.

Mean, median, and standard deviation were used to describe continuous variables. True-positive fluorescent sample (TPFS) was defined as the samples obtained in FL (+) intraoperatively and then confirmed the MGs by histology postoperatively (including gliosis or tumor cell infiltration on histology). True-negative nonfluorescent sample (TNFS) was defined as the samples obtained in FL (−) intraoperatively and then the non-MG tissues were confirmed by histological analysis postoperatively. Sensitivity was defined as the ratio of TPFS and all samples with confirmed MG intraoperatively. Specificity was defined as the ratio of TNFS and all samples that confirmed non-MG histologically. PFS was defined as the time of surgery until MGs was recurrent radiologically or death/last follow-up. Six-month PFS was defined as the PFS at the 6th month after surgery. Overall survival (OS) was defined as the time between the first surgery until death or last follow-up. PFS and OS were employed by Kaplan-Meier means using GraphPad Prism 7.0 software (GraphPad, La Jolla, CA, USA).

## 3. Results

Thirty-eight consecutive patients (19 male and 19 female, median age 50.3 years, range 26 to 71 years) were screened for participation from January 2014 to December 2016 in our series. Two diffuse astrocytomas (WHO grade II) with partial anaplastic astrocytomas (WHO grade III), 1 gemistocytic astrocytoma with partial anaplastic astrocytomas (WHO grade III), 3 oligodendrogliomas (WHO grade III), 4 anaplastic astrocytomas (WHO grade III), and 28 GBMs (WHO grade IV) were obtained by pathology (summarized in Table 1).

**Figure 1:**
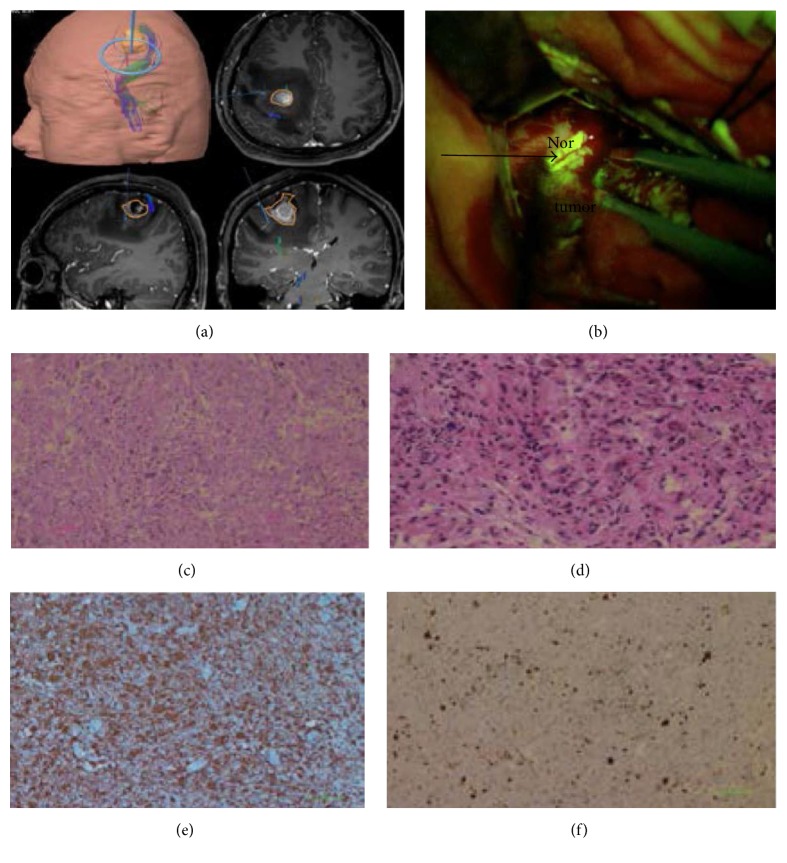
Case (30). (a) Intraoperative imaging based on T1-weighted gadolinium-enhancement MRI navigation during resection of a left frontal GBM. Non-contrast-enhancement region (NCE, blue line) in margin of tumor was obtained according to the navigation indicated. At the same time, the same region (NCE) presented high fluorescence yellow color ((b) black arrow) under the YELLOW 560 filter, and it is feasible to visualize the area of tumor ((b) tumor) that needed to be resected ((b) black arrow and tumor) and distinguished from the normal region ((b) Nor). Tumor was confirmed as GBM ((c) under microscope X 100) by pathology postoperatively. Biopsy specimen was obtained from NCE region ((b) black arrow), pathology was employed postoperatively and confirmed as gliocyte proliferation (under microscope (d) HE-stained ×200, (e) GFAP-stained ×100, and (f) Ki 67-stained ×100).

**Figure 2:**
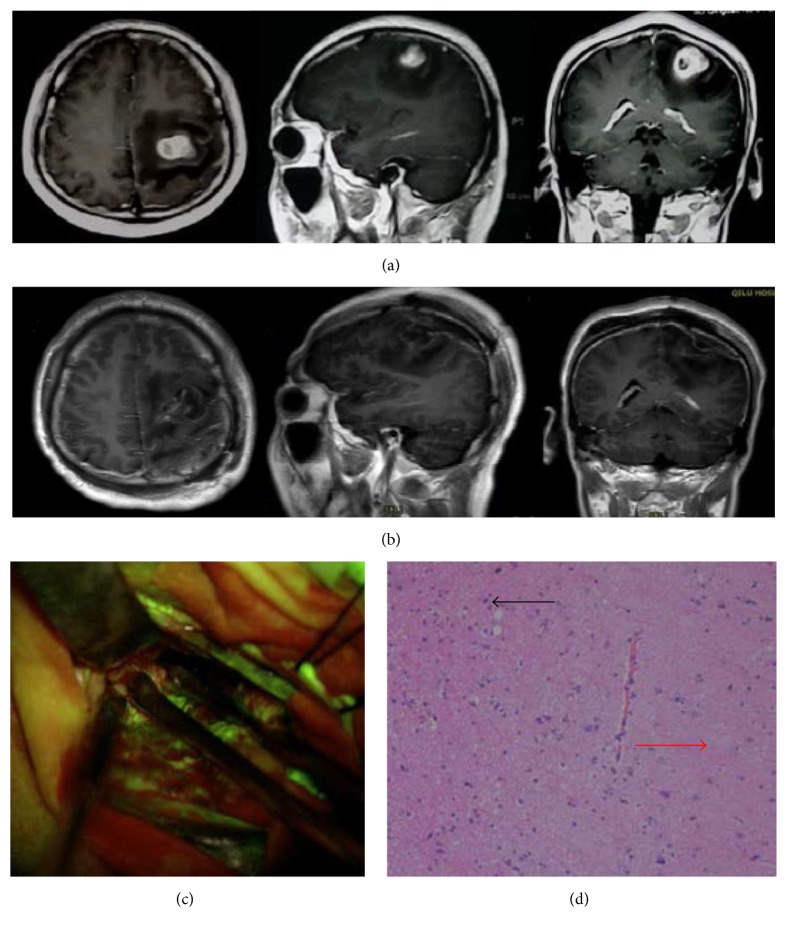
Case (30). (a) Preoperative axial, sagittal, and coronal T1-weighted gadolinium-enhancement MR image revealed a left frontal GBM (tumor volume 13.32 cm^3^). (b) Postoperative (in 24 hours after surgery) axial T1-weighted gadolinium-enhancement MR image, revealing a gross total resection of the tumor without any area of CE region. (c) intraoperative view under YELLOW 560 filter revealing biopsy specimen was obtained beyond the margin of FL-labeled area. (d) Pathological image results revealed almost normal brain tissues (red arrow) and only a few gliocyte proliferation cells (black arrow) were obtained from the region beyond margin of the FL-labeled area (c).

The FL dye passes through the broken BBB and accumulated in the extracellular space of MGs. Based on this mechanism, the pathological tissue of MGs could be visualized the same as the mechanism of gadolinium-enhancement MRI when performing before operation. After induction of anesthesia and before skin incision, FL was injected intravenously. We used 3 mg/kg FL for the first 28 cases and 2 mg/kg FL for the other 10 cases and obtained homologous imaging (Figures 1 and 2). During the time period of craniotomy or dural opening, about 20–40 mins after the administration, we believe that the FL was accumulated in the tumor tissues and did not leak in the normal cerebral areas. After craniotomy, under the YELLOW 560 nm filter, the dura was enhanced with FL in all the cases. Tumor tissue presented bright yellow fluorescence, especially in the CE region according to the neuronavigation, while necrotic part of the tumor appeared “low” or “none” fluorescence. The liquid inside the cyst of the tumor in some cystic cases also was dyed brightly by FL and was brighter than the intraventricular fluid that was not involved in the tumor tissues, even though normal intraventricular fluid was presented with some special fluorescence. YELLOW 560 modules of Pentero 900 microscope could be employed conveniently to discriminate between the fluorescent and nonfluorescent tissues even though at very low-dose levels. Most of the surgery time procedure could be performed under the filter, and with the handgrip switch button, the time could be saved obviously for switching between white light and fluorescence mode during tumor removal.

**Figure 3:**
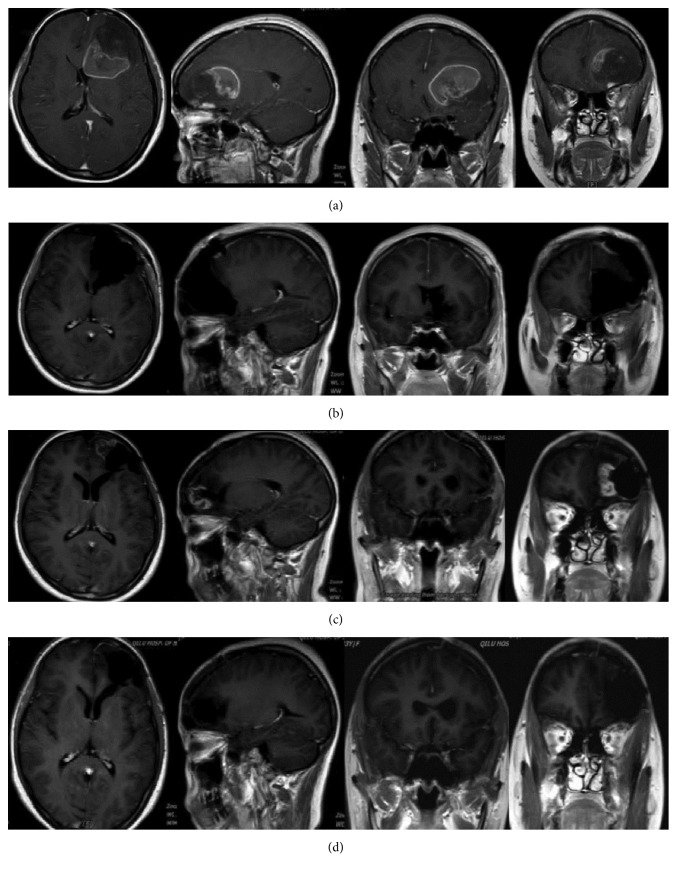
Case (6). (a) Preoperative axial, sagittal, and coronal T1-weighted gadolinium-enhancement MR image revealed a left frontal GBM (tumor volume 51.3 cm^3^). (b) Postoperative (in 72 hours after surgery) axial, sagittal, and coronal T1-weighted gadolinium-enhancement MR image revealed a gross total resection of tumor without any area of CE region. (c) Follow-up axial, sagittal, and coronal T1-weighted gadolinium-enhancement MR image that was obtained 24 months after surgery revealing tumor recurrence. (d) Postoperative (in 72 hours after surgery) axial, sagittal, and coronal T1-weighted gadolinium-enhancement MR image revealed a gross total resection of tumor without any area of CE region.

**Figure 4:**
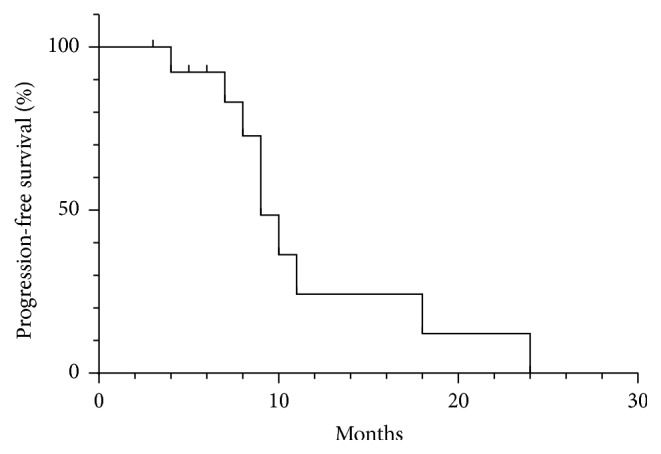
Kaplan-Meier curve of progression-free survival.

Median tumor volume was 64.7 cm^3^ (range 6.0–386.0 cm3) based on preoperative MRI. 35 cases (92.1%) (Figures 2 and 3 and Table 1) were completely removed as all CE regions disappeared postoperatively. The extent of tumor resection was 90.4% (range 82.9%–99.6%) in the residual tumor cases (Figure 4 and Table 1). Intraoperatively, 89 random biopsies (54 FL [+], 35 FL [−]) were obtained both in and out of tumor margins according to FL-guidance. The sensitivity and specificity of FL were 94.4% and 88.6%, respectively (51 TPFS and 31 TNFS), regardless of radiographic localization. Then we choose cases with superficial tumor of the lobes and without invasion of ventricles of brain and releasing of the cerebrospinal fluid during surgery to study FL in CE and NCE region based on T1 weighted MRI. These cases have less possibility with brain shift. Nine cases (cases (13), (15)–(21), and (27)) that have registered in neuronavigation were chosen. We randomly choose pathological biopsies in the MRI-T1 CE and NCE regions. Forty-two biopsies were obtained, including 28 FL[+], 14 FL[−]. Fourteen FL[−] biopsies were obtained from NCE region based on T1 weighted MRI. Of the 28 FL[+] biopsies, there are some biopsies from CE region and some from NCE region. Eighteen FL[+] biopsies (18/28 FL[+]) were obtained from CE region based on T1 weighted MRI and they were all confirmed as tumor tissue. Ten FL[+] biopsies (10/28 FL[+]) including 2 MGs, 7 gliosis or tumor cell infiltrations, and 1 normal cerebral tissues on histology were obtained from NCE region based on T1 weighted MRI and they showed “low” or “high” fluorescence under the microscope (Figure 1 and Table 1).

No side effects of fluorescein administration were observed in the patients. Three epileptic seizure cases were observed during the early postoperative period (during 1 month after surgery) and were considered to be involved with preoperative morbid state of the patients (case (11)).

**Figure 5:**
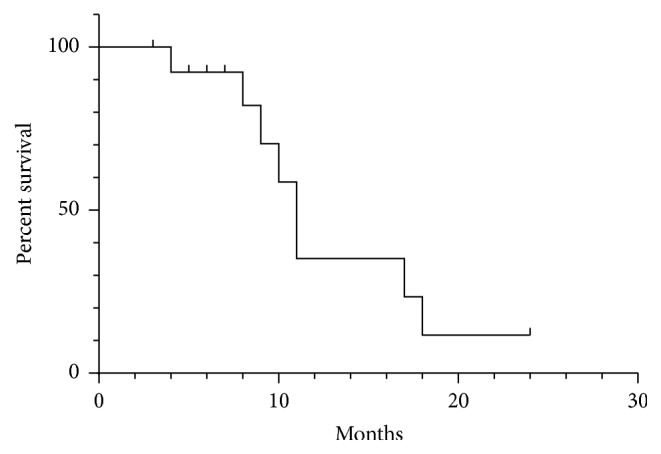
Kaplan-Meier curve of overall survival.

Thirty-five patients (92.1%, 35/38) were followed up; 3 patients (case (7), (14), and (21)) were lost to follow-up. Completion of Stupp protocol was achieved in twenty-three patients (23/35) postoperatively. Seven cases of WHO grade III and 2 cases with recurrent GBM were excluded. The cases that have accepted the initial treatment and complete completion of radiotherapy and chemotherapy and completion of Stupp protocol were 14 cases. A median follow-up of 10.1 months (range 3–24 months) was obtained, and the 6-month PFS was 92.3% (81.2%–91.1%) (Figure 4). During the follow-up period, eight patients died due to progressive tumors (7 cases, cases (2), (3), (5), (9), (15), (16), and (18)) or uncorrelated factors (such as severe pneumonia in one case, case (1)). The calculated median survival was 11 months (Figure 5).

## 4. Discussion

Safe maximal resection is an essential predictor for preferable prognosis and lower recurrence of MGs, but precisely total resection of tumor is still a great challenge due to the infiltrated character of MGs. Improvement of the microscope in neurosurgery and choosing a reasonable treatment strategy for MGs are extremely important factors. After first attempt to explore the novel label for intracranial tumors by Moore et al. [9, 10], they resected 46 intracranial tumors under ordinary illumination, then examined the residual cavity under the ultraviolet lamp, and consequently obtained only a rough assessment result of FL valuation for brain tumor diagnosis. Murray ameliorated the procedure and employed a special arc-lighting system that concatenated with the surgical headlight to resect the fluorescein-stained tissues under dynamic conditions [22]. By both techniques, because of the light source system and the imaging system disadvantages, none of these produced satisfactory fluorescent imaging results especially in the boundary of tumor-normal tissues. Kuroiwa employed FL-guided neurosurgery for 10 MGs using a dose of 8 mg/kg bodyweight under the microscope including two sections of insertion and obtained clearer image compared to the above both groups and totally resected 80% (8/10 cases) of the intracranial tumors according to the postoperative CT/MRI enhancement. However, the group had never developed any advanced special microscope with special filter for FL-guided surgery. A modern surgical microscope of PENTERO 900 (Carl Zeiss Meditec) with a new special barrier filter (YELLOW 560 nm), designed according to the characters of fluorescein dye with a peak excitation at 465 to 490 nm and emission between 500 and 550 nm, had been employed for FL-guided surgery with very low dosage (3-4 mg/kg bodyweight) by Schebesh et al. They reported their first experience with 35 heterogeneous patients with total resection of 80% according to the analysis of volumetric data, which meant a gross total resection of >95% of the contrast-enhancing tumor, but no further details were reported [13]. Acerbi et al. noted that the new filter with YELLOW 560 nm could supply a higher specificity to the filter characteristics, a better delineation of the fluorescent dyed tissues and a continuous fluorescent condition compared to YELLOW 400 (Pentero with Fluorescence Kit, Carl Zeiss, Germany). Using FL-guided surgery, 12 glioblastomas were completely removed in 75% of the patients according to the volumetric analysis at a dose of 5 mg/kg bodyweight under the YELLOW 560 nm surgical microscope filter compared to 10 mg/kg bodyweight under YELLOW 400 [12]. In their prospective phase II trial (FLUOGLIO) in 2014, a gross total removal was achieved in 80% of 20 consecutive patients with high-grade gliomas (HGGs) based on volumetric analysis; sensitivity of 94% and specificity of 89.5%, respectively, were also reported. And this helped them to make further efforts, which showed a 6-month PFS rate of 71.4% and 11-month median survival [16]. Recently, at a dose of 3 mg/kg bodyweight, Diaz et al. reported 12 patients who underwent resection of MGs guided by FL. The results showed total resection of 100%, sensitivity of 82.2%, and specificity of 90.9%, respectively [18]. Justin A obtained total removal of 93.1% at the same dose and sensitivity of 87.9% who underwent aggressive resection facilitated by FL-guidance for GBMs or recurrent GBMs. No specificity was calculated because of the lack of TNS in their study. In our series, all 38 patients who were suffering with MGs underwent FL-guided surgery under the YELLOW 560 nm surgical microscope filter. FL enhancement was observed unambiguously in all MGs depending on the preoperative MRI-contrast-enhancement regions and intraoperative neuronavigation; even though the dose was reduced to 2 mg/kg bodyweight, the results showed advantages of YELLOW 560 nm filter. Consequently, GTR of 92.1% (35/38) was accomplished according to our aggressive resection strategy and volume analysis; even in the residual tumor cases the tumor removal rate achieved 90.4% in volume (range 82.9%–99.6%). Regardless of neuronavigation based on T1 weighted MRI, the sensitivity and specificity of FL could be calculated as 94.4% and 88.6%, respectively. No side effects and adverse reactions related to FL administration were observed, though anaphylactic reactions had been reported in literature related to higher doses [14, 15].

Preoperative gadolinium-enhancement MRI was considered to be the BBB disruption label, which also has been the fundamental strategy for GTR of MGs depending on postoperative gadolinium-enhancement MRI till now. But even 2 cm region outside the margin of MGs resulted in the recurrence and poor prognosis rates and attributed to the infiltrated character of MGs, which had been identified by many studies [6, 23, 24]. FL is considered to be another BBB disruption label that especially acts as an intraoperative real-time visualization agent. This had been supported by several previous studies [6, 23, 24] and identified in in vitro and in vivo models by Diaz et al. [18]. In our study, FL-guided surgery when combined with neuronavigation (BrainLab) produced fluorescein staining of 100% not only in CE regions, identified by pathological biopsies, but also in the NCE regions (10/28). The phenomenon may be related to the differential permeability profiles of fluorescein and gadolinium according to different molecular sizes and pathological correlation of tumors [18]. Furthermore, the “gliosis or tumor cell infiltration” in pathological diagnosis was obtained in the margin outside CE regions, which may result in the recurrence and poor prognosis and was classified as “negative” in previous studies [18]. In the largest nonvolumetric studies regarding the extent of resection of high-grade gliomas [23], GTR showed significant survival advantages compared to the near-total resection and additional resection of NCE regions, which in turn may contribute to a significant added survival advantage. Above all, fluorescein stained region could be including and larger than the CE region; residual tumors in both might result in the recurrence and poor prognosis. Thus, safe and aggressive or super-total resection of MGs should be evolved in order to achieve safe and maximal resection, which could be deemed to the reasonable treatment strategy for MGs. Neuronavigation depended on the preoperative imaging plan, which provided neuroanatomy information during operation, but its disadvantages especially when being used solely, such as brain shift, accurate preoperative registration, and reliance on preoperative enhancement to identify tumor boundary, impeded real-time guiding resection of tumors intraoperatively. In our experience, combining neuronavigation with the standard procedure under the YELLOW 560 nm surgical microscope filter, aggressive resection could be safe and reliable, which in turn is facilitated by FL-guidance. Thus, we also paid attention to the NCE regions of T1 weighted MRI, and mostly FL-stained biopsies showed “low” fluorescence in NCE regions (10/28 FL[+]) but confirmed 2 MGs and 7 gliosis or tumor cell infiltrations on pathology (Figure 1), even though 1 was confirmed as normal cerebral tissue at the margin of the tumor. However, it should not be ignored regarding the fact that the mechanism of FL accumulation in the intercellular space was related to the passage through a broken BBB, and not as label of high-grade glial cells, just as our investigation in NCE regions. Nonetheless, this cellular reduction procedure on our aggressive resection purpose may still further the progress due to its larger region than the CE region and included more MG cells, which may result in the recurrence, though FL-labeled region may not include all the cells of MGs.

Our median follow-up period of 10.1 months was short term duration and still limited to a retrospective study though we have the strict inclusion and exclusion criteria according to the FLUOGLIO. Thus, it was not sufficient to come to a final conclusion based on long-term outcomes, but our preliminary results showed 92.3% estimated 6-month PFS without disease progression. Furthermore, our data showed even higher PFS (92.3% versus 71.4%) and similar median survival (11 months versus 11 months) rates compared with FLUOGLIO data. The reason might be due to the inclusion of many complicated factors, and one such is the intraoperative aggressive resection strategy used, which in turn may reduce more MG cells and decrease the potential risk of tumor recurrence though the exact reason was not precisely included in our study.

Published protocols about 5-ALA-guidance, including a randomized controlled multicentre phase III trial by Stummer et al. [6], described as a time-consuming procedure, should be administrated preoperatively (before 3–6 hours), and patients should also be shielded from sunlight postoperatively for 48-hour period. The best timing of i.v. FL before resection was also considered in the previous studies [12, 13]. In our experience, after the anesthesia induction, 2 mg/kg bodyweight FL (10%) was injected intravenously and was left to note an appropriate permitting time to discriminate the tumors and brain tissues. Furthermore, under Pentero 900, the time of operation had also been saved in order to obtain navigation useful period of FL-guidance due to its convenient to switch back and forth between white light and the filter apparatus. FL requires significantly less time from the time of administration for visualization compared with 5-ALA [11]. Because of these advantages, FL-guidance may be more suitable for extensive promotion especially during emergency operations compared to 5-ALA guidance. The extensive use of this new technology should also balance between the safety and efficacy of available interventions and the current socioeconomic milieu of treatment strategy for MGs. 5-ALA is available as an approved drug for the malignant glioma [6]. The phase II clinical trials were only done for FL [12]. However, the FL has been used for brain tumor biopsy in early years, so there is no problem in security of FL [9, 10]. Our results have been helpful for the accumulation of FL success clinical materials in the application of malignant glioma. 5-ALA costs approximately 950€ for one application, whereas FL costs 25€ only [13]. Special equipment (surgical microscope filter) for 5-ALA is also more expensive compared with the YELLOW module.

## 5. Conclusion

In conclusion, FL-guided neurosurgery was based on the mechanism of accumulated FL in the intercellular space through broken BBB. This when combined with T1 weighted contrast-enhancement neuronavigation was proved to be safe and effective in the resection of MGs. Under the strategy of aggressive resection, it was feasible to achieve cell reduction resection based on postoperative T1 weighted CE MRI, which was further confirmed by pathology and may obtain preferable prognosis though there was no control group to compare in our study.

## Figures and Tables

**Table 1:** Clinical characteristics in summary.

Number	Age/sex	Symptoms/signs	Localization	Tumor size (cm^3^)	Pathology	% of resection	Number of biopsies
(1)	67F	Headache, aphasia	LT/P/O	179.52	GBM	100	1
(2)	47M	Seizure, somnolence	LT/P/O	70.56	GBM	100	1
(3)	38M	Headache, dizziness, blurred vision	LP/O	18	GBM	100	1
(4)	57M	Headache, dizziness	RF	40.18	GBM	100	1
(5)	30F	Headache, vomit	RTP	50.975	GBM	100	1
(6)	32F	Headache	LF/T	51.324	GBM	100	1
(7)	61M	Headache, dizziness	RT/O	23.161	GBM	100	1
(8)	51M	Headache, vomit, somnolence	BF	62.54	GBM	100	1
(9)	64F	Headache, dizziness	RF	65.52	GBM	100	1
(10)	42M	Aconuresis, IICP	RF	221.4	GBM	100	1
(11)	42M	Seizure	RF	15.732	AA (WHO III)	100	4
(12)	71F	Headache, somnolence	RT/P	126.759	GBM	100	2
(13)	49M	Recurrent	RF	26.4	DA (partial AA, WHO III)	100	7
(14)	62F	Aphasia, right prosopoplegia	LF	68.04	GBM	100	3
(15)	48F	Headache, left hemiparesis	LF/T/I	84.48	GBM	100	5
(16)	66M	Headache, somnolence	LP/O	122.4	GBM	88.6	6
(17)	36F	Headache, IICP	RF/T	28.7	GA (partial AA, WHO III)	100	2
(18)	50F	Headache, left hemiparesis, IICP	RF	37.44	GBM	100	2
(19)	71F	Left hemiparesis	LF/P	36	GBM	100	5
(20)	49M	Seizure	RF	70.119	OD (WHO III)	100	5
(21)	35M	Seizure	LF	49.02	OD (WHO III)	100	2
(22)	49F	Recurrent	RF/T/I	94.875	rGBM	99.6	4
(23)	58M	Seizure	LF	6.048	GBM	100	1
(24)	41F	Headache, aphasia	LF/T/P	81.567	AA (WHO III)	100	3
(25)	61F	Seizure	RF/T/I	21.06	DA (partial AA, WHO III)	100	2
(26)	26F	Seizure	LF	44	OD (WHO III)	100	4
(27)	34F	Recurrent	LF	13.888	rGBM	100	8
(28)	45M	Seizure, left hemiparesis	RP	36	AA (WHO III)	100	2
(29)	42F	Nausea, IICP	RT	30.636	AA (WHO III)	100	1
(30)	32M	Seizure, right tendon hyperreflexia	LF	13.32	GBM	100	3
(31)	63M	Headache, somnolence	RF	23.826	GBM	100	1
(32)	65M	Headache, somnolence	LF	28.35	GBM	100	1
(33)	62F	Headache, somnolence	LF/T	40.572	GBM	100	1
(34)	46M	Headache, coma	RT/P/O	80.276	GBM	82.9	1
(35)	46M	Recurrent	RT/P/O	385.92	GBM	100	1
(36)	61M	Memory deterioration, visual field defect	LT	56.925	GBM	100	1
(37)	56F	Headache	RP	19.448	GBM	100	1
(38)	56F	Headache, left hemiparesis	RPO	32.2	GBM	100	1

F: female, M: male, L: left, R: right, T: temporal lobe, P: parietal lobe, O: occipital lobe, I: insular lobe, GBM: glioblastoma multiforme, rGBM: recurrent glioblastoma multiforme, AA: anaplastic astrocytoma, DA: diffuse astrocytomas, OD: oligodendrogliomas, and GA: gemistocytic astrocytoma.

## References

[1] Orringer D., Lau D., Khatri S. (2012). Extent of resection in patients with glioblastoma: Limiting factors, Perception of resectability, and effect on survival. *Journal of Neurosurgery*.

[2] Salvati M., Pichierri A., Piccirilli M. (2012). Extent of tumor removal and molecular markers in cerebral glioblastoma: A combined prognostic factors study in a surgical series of 105 patients - Clinical article. *Journal of Neurosurgery*.

[3] Wirtz C. R., Albert F. K., Schwaderer M. (2000). The benefit of neuronavigation for neurosurgery analyzed by its impact on glioblastoma surgery. *Neurological Research*.

[4] Unsgaard G., Ommedal S., Muller T. (2002). Neuronavigation by intraoperative three-dimensional ultrasound: initial experience during brain tumor resection. *Neurosurgery*.

[5] Shinoda J., Yano H., Yoshimura S.-I. (2003). Fluorescence-guided resection of glioblastoma multiforme by using high-dose fluorescein sodium. Technical note. *Journal of Neurosurgery*.

[6] Stummer W., Pichlmeier U., Meinel T., Wiestler O. D., Zanella F., Reulen H.-J. (2006). Fluorescence-guided surgery with 5-aminolevulinic acid for resection of malignant glioma: a randomised controlled multicentre phase III trial. *The Lancet Oncology*.

[7] Novotny H. R., Alvis D. L. (1961). A method of photographing fluorescence in circulating blood in the human retina.. *Circulation*.

[8] Kwan A. S. L., Barry C., McAllister I. L., Constable I. (2006). Fluorescein angiography and adverse drug reactions revisited: the Lions Eye experience. *Clinical and Experimental Ophthalmology*.

[9] Moore G. E. (1947). Fluorescein as an agent in the differentiation of normal and malignant tissues. *Science*.

[10] Moore G. E., Peyton W. T., French L. A., Walker W. W. (1948). The clinical use of fluorescein in neurosurgery. *Journal of Neurosurgery*.

[11] Koc K., Anik I., Cabuk B., Ceylan S. (2008). Fluorescein sodium-guided surgery in glioblastoma multiforme: A prospective evaluation. *British Journal of Neurosurgery*.

[12] Acerbi F., Broggi M., Eoli M. (2013). Fluorescein-guided surgery for grade IV gliomas with a dedicated filter on the surgical microscope: Preliminary results in 12 cases. *Acta Neurochirurgica*.

[13] Schebesch K.-M., Proescholdt M., Höhne J. (2013). Sodium fluorescein-guided resection under the YELLOW 560 nm surgical microscope filter in malignant brain tumor surgery - A feasibility study. *Acta Neurochirurgica*.

[14] Dilek O., Ihsan A., Tulay H. (2011). Anaphylactic reaction after fluorescein sodium administration during intracranial surgery. *Journal of Clinical Neuroscience*.

[15] Tanahashi S., Iida H., Dohi S. (2006). An anaphylactoid reaction after administration of fluorescein sodium during neurosurgery [25]. *Anesthesia and Analgesia*.

[16] Acerbi F., Broggi M., Eoli M. (2014). Is fluorescein-guided technique able to help in resection of high-grade gliomas?. *Neurosurgical Focus*.

[17] Stupp R., Mason W. P., van den Bent M. J. (2005). Radiotherapy plus concomitant and adjuvant temozolomide for glioblastoma. *The New England Journal of Medicine*.

[18] Diaz R. J., Dios R. R., Hattab E. M. (2015). Study of the biodistribution of fluorescein in glioma-infiltrated mouse brain and histopathological correlation of intraoperative findings in high-grade gliomas resected under fluorescein fluorescence guidance. *Journal of Neurosurgery*.

[19] Neira J. A., Ung T. H., Sims J. S. (2017). Aggressive resection at the infiltrative margins of glioblastoma facilitated by intraoperative fluorescein guidance. *Journal of Neurosurgery*.

[20] Cordella R., Acerbi F., Broggi M. (2013). Intraoperative neurophysiological monitoring of the cortico-spinal tract in image-guided mini-invasive neurosurgery. *Clinical Neurophysiology*.

[21] Wen P. Y., Macdonald D. R., Reardon D. A. (2010). Updated response assessment criteria for high-grade gliomas: response assessment in neuro-oncology working group. *Journal of Clinical Oncology*.

[22] Murray K. J. (1982). Improved surgical resection of human brain tumors: Part 1. A preliminary study. *Surgical Neurology*.

[23] Li Y. M., Suki D., Hess K., Sawaya R. (2016). The influence of maximum safe resection of glioblastoma on survival in 1229 patients: Can we do better than gross-total resection?. *Journal of Neurosurgery*.

[24] Silbergeld D. L. (2008). Extent of resection and survival in glioblastoma multiforme: Identification of and adjustment for bias - Commentary. *Neurosurgery*.

