# Corrigendum to “Sodium Fluorescein-Guided Resection under the YELLOW 560 nm Surgical Microscope Filter in Malignant Gliomas: Our First 38 Cases Experience”

**DOI:** 10.1155/2018/6348625

**Published:** 2018-05-14

**Authors:** Ningning Zhang, Hailong Tian, Dezhang Huang, Xianbing Meng, Wenqiang Guo, Chaochao Wang, Xin Yin, Hongying Zhang, Bin Jiang, Zheng He, Zhigang Wang

**Affiliations:** Department of Neurosurgery, Qilu Hospital, Shandong University, Qingdao, China

In the article titled “Sodium Fluorescein-Guided Resection under the YELLOW 560 nm Surgical Microscope Filter in Malignant Gliomas: Our First 38 Cases Experience” [1], there was an error in the authors' affiliation. The corrected affiliation is shown above.
